# Supplementary material for: Prevalence of ADHD in Accident Victims: Results of the PRADA Study
Source: J Clin Med. 2019 Oct 8;8(10):1643. doi: 10.3390/jcm8101643 (PMC6832520; doi:10.3390/jcm8101643)
Supplement: Supplementary file 1 [file jcm-08-01643-s001.zip › Accident Questionnaire(translated)_SKS.docx]

**PRADA Study – Course of accident**

Department: __________ Patient-ID: __________ Date: __________

*Note: This questionnaire should be filled in by the study personnel by asking the patient specifically.*

1. What happened? Please describe the course of the accident in one sentence.
2. Where did the accident occur?
3. In road traffic
4. At home
5. When exercising/doing sport
6. Workplace/place of education
7. Public place. *Where exactly?*
8. Others. *Where exactly?*
9. If the accident occurred **on the road**: Which means of transport did you use?
10. Public transportation
11. Car
12. Motorcycle/scooter
13. Bicycle
14. Scooters/roller skates/Inline skates/skateboard
15. On foot/as pedestrian
16. Other. *Which one exactly?*
17. If the accident occurred **at home**: What have you been doing?
18. Everyday activities (showering, cooking, climbing stairs, ...). Please specify more precisely, if yes.
19. Home crafting
20. In rest/sitting/lying
21. Others. *Please specify.*
22. If the accident occurred **during sport**: Which sport have you been doing?
23. Jogging
24. Ball sports. *Please specify.*
25. Cycling *(here only sportive cycling is meant!)*
26. Swimming/other water sports
27. Winter sports. *Please specify.*
28. Combat sports
29. Climbing
30. Athletics
31. Others. *Please specify.*
32. If the accident occurred **at work or place of education**: Where did the accident took place?
33. At work
34. At school
35. At university
36. Did you cause the accident yourself?
37. Yes
38. No (another person is to blame/no one is to blame)
39. Were you under the influence of substances *(alcohol and 'illegal' drugs ONLY)* during the accident?
40. Yes
41. No
42. **If yes**, what substance was it?
43. Alcohol
44. Other drugs. *Which one?*
45. Did you take any **medication** on the day of the accident (before the accident) **that you would otherwise NOT have taken**?
46. Yes
47. No
48. **If yes**, what medication was it?
49. Sleeping medication/sedatives
50. Other psychiatric drugs
51. Cardiovascular drugs
52. Anticoagulants
53. Painkillers *(especially opioids)*
54. Stimulants
55. Other. *Which one?*
56. Do you **regularly** take medication?
57. Yes
58. No
59. **If yes**, which daily medication do you take?
60. Sleeping medication/sedatives
61. Other psychiatric drugs
62. Cardiovascular drugs
63. Anticoagulants
64. Painkillers (especially opioids)
65. Stimulants
66. Other. *Please specify.*
67. Were you **distracted** shortly before or during the accident? Because of…
68. Mobile phone/smartphone use
69. Conversation with another person
70. Environment/surroundings
71. Headphones/music
72. Other. *Through what, exactly?*
73. Are you feeling stressed **right now**? If so, what is the reason?
74. Yes
75. No
76. Were you stressed or emotionally aroused **before/during the accident**?
77. Yes
78. No
79. Was there any emotional or stressful **event before the accident**? If so, what kind of event?
80. Argument/conflict
81. Stressful/pressurizing situation (e.g. stress at work/at university/...)
82. Bereavement (e.g. death of a close person)
83. Other. *What exactly?*
84. Were you involved in **other accidents** in the past year?
85. Yes, briefly state what happened.
86. No
87. Do you have any medical history?
88. Yes
89. No
90. **If yes**, which disease(s) do you suffer from?
91. Affective disorder (depression, bipolar disorder)
92. Personality disorder
93. Eating disorder
94. Anxiety disorder
95. Schizophrenia
96. Substance abuse/dependence
97. ADHD, diagnosed in the past
98. Diseases of the skeletal and musculoskeletal system *(especially orthopaedic diseases; NOT the current trauma)*
99. Cardiovascular diseases
100. Thyroid diseases
101. Metabolic diseases (D.m., obesity, ...)
102. Neurological disorders. Which one?
103. Chronic pain
104. Other. *Which ones exactly?*
105. Did you overestimate your abilities regarding the accident or have you been overconfident?
106. Yes
107. No
108. Did you suffer from lack of sleep or fatigue before or during the accident?
109. Yes
110. No
