# Supplementary material for: Prevalence of ADHD in Accident Victims: Results of the PRADA Study
Source: J Clin Med. 2019 Oct 8;8(10):1643. doi: 10.3390/jcm8101643 (PMC6832520; doi:10.3390/jcm8101643)
Supplement: Supplementary file 1 [file jcm-08-01643-s001.zip › Supplemental_Table1.docx]

Supplemental Table 1: Place and circumstances of accidents

|  |  |  |  |  |
| --- | --- | --- | --- | --- |
| **Road traffic** | **ADHD (n)** | **%** | **Controls (n)** | **%** |
| Public transport | 1 |  | 2 |  |
| Car | 4 |  | 13 |  |
| Motorbike | 5 |  | 21 |  |
| Bicycle | 3 |  | 17 |  |
| Scooters/roller skates/Inline skates/skateboard | 1 |  | 1 |  |
| Pedestrian | 6 |  | 10 |  |
| Other | 0 |  | 0 |  |
| **Total Road traffic accidents** | **20** | **35.71** | **64** | **29.90** |
|  |  |  |  |  |
| **At home** | **ADHD (n)** | **%** | **Controls (n)** | **%** |
| Daily activity (showering. cleaning…) | 1 |  | 13 |  |
| Home crafting | 3 |  | 4 |  |
| Resting | 1 |  | 0 |  |
| Other activity at home | 2 |  | 3 |  |
| **Total home accidents** | **7** | **12.5** | **20** | **9.35** |
|  |  |  |  |  |
| **Sports** | **ADHD (n)** | **%** | **Controls (n)** | **%** |
| Running | 0 |  | 0 |  |
| ball sports | 1 |  | 8 |  |
| cycling | 0 |  | 3 |  |
| swimming | 0 |  | 0 |  |
| winter sports | 3 |  | 15 |  |
| martial arts | 1 |  | 0 |  |
| climbing | 0 |  | 0 |  |
| athletics | 1 |  | 1 |  |
| other | 2 |  | 9 |  |
| **Total sport accidents** | **8** | **14.23** | **36** | **16.82** |
|  |  |  |  |  |
|  | **ADHD (n)** | **%** | **Controls (n)** | **%** |
| Work | 10 |  | 62 |  |
| School | 0 |  | 2 |  |
| University | 0 |  | 0 |  |
| **Total work-related accidents** | **10** | **17.86** | **64** | **29.91** |
|  |  |  |  |  |
